# Can emotional intelligence be improved? A randomized experimental study of a business-oriented EI training program for senior managers

**DOI:** 10.1371/journal.pone.0224254

**Published:** 2019-10-23

**Authors:** Raquel Gilar-Corbi, Teresa Pozo-Rico, Bárbara Sánchez, Juan-Luís Castejón

**Affiliations:** Developmental and Educational Psychology Department, University of Alicante, San Vicente del Raspeig, Alicante, Spain; Universidad Nacional de Educacion a Distancia (UNED), SPAIN

## Abstract

Purpose: This article presents the results of a training program in emotional intelligence. Design/methodology/approach: Emotional Intelligence (EI) involves two important competencies: (1) the ability to recognize feelings and emotions in oneself and others, and (2) the ability to use that information to resolve conflicts and problems to improve interactions with others. We provided a 30-hour Training Course on Emotional Intelligence (TCEI) for 54 senior managers of a private company. A pretest-posttest design with a control group was adopted. Findings: EI assessed using mixed and ability-based measures can be improved after training. Originality/value: The study’s results revealed that EI can be improved within business environments. Results and implications of including EI training in professional development plans for private organizations are discussed.

## Introduction

This research study focused on EI training in business environments. Accordingly, the aim of the study was to examine the effectiveness of an original EI training program in improving the EI of senior managers. In this article, we delineate the principles and methodology of an EI training program that was conducted to improve the EI of senior managers of a private company The article begins with a brief introduction to the main models of EI that are embedded with the existing scientific literature. This is followed by a description of the EI training program that was conducted in the present study and presentation of results about its effectiveness in improving EI. Finally, the present findings are discussed in relation to the existing empirical literature, and the limitations and conclusions of the present study are articulated.

### Defining EI

Various models of emotional intelligence (EI) have been proposed. The existing scientific literature offers three main models of EI: mixed, ability, and trait models. First, mixed models conceptualize EI as a combination of emotional skills and personality dimensions such as assertiveness and optimism [1, 2]. Thus, according to the Bar-On model [3], emotional-social intelligence (ESI) is a multifactorial set of competencies, skills, and facilitators that determine how people express and understand themselves, understand and relate to others, and respond to daily situations The construct of ESI consists of 10 key components (i.e., self-regard, interpersonal relationships, impulse control, problem solving, emotional self-awareness, flexibility, reality-testing, stress tolerance, assertiveness, and empathy) and five facilitators (optimism, self-actualization, happiness, independence, and social responsibility). Emotionally and socially intelligent people accept and understand their emotions; they are also capable of expressing themselves assertively, being empathetic, cooperating with and relating to others in an appropriate manner, managing stressful situations and changes successfully, solving personal and interpersonal problems effectively, and having an optimistic perspective toward life. Second, ability models of EI focus on the processing of information and related abilities [3]. Accordingly, Mayer and Salovey [4] have conceptualized EI as a type of social intelligence that entails the ability to manage and understand one’s own and others’ emotions. Indeed, this implies that EI also entails the ability to use emotional information to manage thoughts and actions in an adaptive manner [5]. Third, the trait EI approach understands EI as emotion-related information [6]. According to trait models, EI refers to self-perceptions and dispositions that can be incorporated into fundamental taxonomies of personality. Therefore, according to Petrides and Furnham [7], trait EI is partially determined by several dimensions of personality and can be situated within the lower levels of personality hierarchies. However, it is a distinct construct that can be differentiated from other personality constructs. In addition, the construct of trait EI includes various personality dispositions as well as the self-perceived aspects of social intelligence, personal intelligence, and ability EI. The following facets are subsumed by the construct of trait EI: adaptability, assertiveness, emotion perception (self and others), emotion expression, management (others), and regulation, impulsiveness (low), relationships, self-esteem, self-motivation, social awareness, stress management, trait empathy, happiness, and optimism [7]. Finally, as Hodzic et al. [8] have indicated, most existing definitions of EI permit us to draw the conclusion that EI is a measurable individual characteristic that refers to a way of experiencing and processing emotions and emotional information. It is noteworthy that these models are not mutually exclusive [7].

### Effects of EI on different outcomes

EI has been found to be related to workplace performance in highly demanding work environments (see e.g. [9]). Consequently, companies, entities, and organizations tend to recognize the importance of EI, promote it on a daily basis to facilitate career growth, and recruit those who possess this ability. [10].

With regard to research that has examined the EI-performance link, Van Rooy and Viswesvaran [11] conducted a metanalytic study to examine the predictive power of EI in the workplace. They found that approximately 5% of the variance in workplace performance was explained by EI, and this percentage is adequately significant to increase savings and promote improvements within organizations. In addition, the authors concluded that further in-depth investigations are needed to comprehensively understand the construct of EI.

However, the EI-performance link must be interpreted with caution. Specifically, Joseph and Newman [12] examined emotional competence in the workplace and found that EI predicts performance among those with high emotional labor jobs but not their counterparts with low emotional labor jobs. In addition, they indicated that further research is required to delineate the relationship between EI and actual job performance, gender and race differences in EI, and the utility of different types of EI measures that are based on ability or mixed models in training and selection. Accordingly, Pérez-González and Qualter [13] have underscored the need for emotional education. Further, Brasseur et al. [14] found that better job performance is related to EI, especially among those with jobs for which interpersonal contact is very important.

It is noteworthy that EI is positively related to job satisfaction. Accordingly, Chiva and Alegre [15] found that there was an indirect positive relationship between self-reported EI (i.e., as per mixed models) and job satisfaction. A total of 157 workers from several companies participated in this study. These findings suggest that people with higher levels of EI are more satisfied with their jobs and demonstrate a greater capacity for learning than their counterparts with lower levels of EI.

Similarly, Sener, Demirel, and Sarlak [16] adopted a mixed model of EI and examine its effect on job satisfaction. They found that individuals with strong emotional and social competencies demonstrated greater self-control. A total of 80 workers participated in this study. They were able to manage and understand their own and others’ emotions in an intelligent and adaptive manner in their personal and professional lives.

In addition, EI (i.e., as per mixed models) predicts job success because it influences one’s ability to deal with environmental demands and pressures [17]. Therefore, it has been contended that several components of EI (i.e., as per mixed models) contribute to success and productivity in the workplace [18]; future research studies should extend this line of inquiry. Several studies have shown that people with high levels of ability EI communicate in an interesting and assertive manner, which in turn makes others feel more comfortable in the workplace [19]. In addition, it has been contended that EI (i.e., as per mixed models) plays a valuable role in group development because effective teamwork occurs when team members possess knowledge about the strengths and weaknesses of others and the ability to use these strengths when necessary [15, 20]. It is especially important for senior managers to demonstrate high levels of EI because they play a predominant role in team management, leadership, and organizational development.

Finally, studies that have examined the relationship between EI and wellbeing have found that ability EI is a predictor of professional success, wellbeing, and socially relevant outcomes [21–23]. Extending this line of inquiry, Slaski and Cartwright [24] investigated the relationship between EI and the quality of working life among middle managers and found that higher levels of EI is related to better performance, health, and wellbeing.

### EI and leadership

The actions of organizational leaders play a crucial role in modulating the emotional experiences of employees [25]. Accordingly, Thiel, Connelly, and Griffith [26] found that, within the workplace, emotions affect critical cognitive tasks including information processing and decision making. In addition, the authors have contended that leadership plays a key role in helping subordinates manage their emotions. In another study, Batool [27] found that the EI of leaders have a positive impact on the stress management, motivation, and productivity of employees.

Gardner and Stough [28] further investigated the relationship between leadership and EI among senior managers and found that leaders’ management of positive and negative emotions had a beneficial impact on motivation, optimism, innovation, and problem resolution in the workplace. Therefore, the EI of directors and managers is expected to be positively correlated with employees’ work motivation and achievement.

Additionally, EI competencies are involved in the following activities: choosing organizational objectives, planning and organizing work activities, maintaining cooperative interpersonal relationships, and receiving the support that is necessary to achieve organizational goals [29]. In this regard, some authors have provided compelling theoretical arguments in favor of the existence of a relationship between EI and leadership [30–34]. In this way, several researches [30–34] show that EI is a core and key variable positively related to effective and transformational leadership and this is important for positive effects on job performance and attitudes that are desirable in the organization.

Further, people with high levels of EI are more capable of regulating their emotions to reduce work stress [35]; thus, it is necessary to emphasize the importance of EI in order to meet the workplace challenges of the 21st century.

In conclusion, EI competencies are considered to be key qualities that individuals who occupy management positions must possess [36]. Further, EI transcends managerial hierarchies when an organization flourishes [37]. Finally, emotionally intelligent managers tend to create a positive work environment that improves the job satisfaction of employees [38].

### EI trainings

Past studies have shown that training improves the EI of students [22, 39, 40–44], employees [45–47], and managers [48–52]. More specifically, within the academic context, Nelis et al. [22] found that group-based EI training significantly improved emotion identification and management skills. In another study, Nelis et al. [39] found that EI training significantly improved emotion regulation and comprehension and general emotional skills. It also had a positive impact on psychological wellbeing, subjective perceptions of health, quality of social relations, and employability. Similarly, several studies that have been conducted within the workplace have shown that EI can be improved through training [45–52] and have underscored the key role that it plays in effective performance [53, 54].

In addition, two relevant metanalyses [8, 55] concluded that there has been an increase in research interest in EI, recognition of its influence on various aspects of people’s lives, and the number of interventions that aim to improve EI. Relatedly, Kotsou et al. [55] and Hodzic et al. [8] reviewed the findings of past studies that have examined the effects of EI training to explore whether such training programs do indeed improve EI.

First, Hodzic et al. [8] concluded that EI training has a moderate effect on EI and that interventions that are based on ability models of EI have the largest effects. In addition, the improvements that had resulted from these interventions were found to have been temporally sustained.

Second, the conclusions of Kotsou et al.’s [55] systematic review of the literature on the effectiveness of EI training make it evident that more rigorous and controlled studies are needed to permit one to draw concrete conclusions about whether training improves ability EI. Studies that had adopted mixed models of EI tended to more consistently find that training improves EI. Accordingly, the results of Kotsou et al.’s [55] metanalytic study revealed that EI training enhances teamwork, conflict management, employability, job satisfaction, and work performance.

Finally, it is necessary to identify and address the limitations of past interventions in future studies to improve their quality and effectiveness.

### Purpose of the study

In the systematic review conducted by Kotsou et al. [55] regarding research published on interventions to improve EI in adults, one out of five studies with managers, was performed on a sample of middle managers, without randomization, with an inactive control group, no immediate measures after the training, and only one evaluation was performed six months after the training. In the other four studies collected in Kotsou et al.’s systematic review [55], only one study utilized a control group (inactive control group), one employed randomizations, and two studies performed follow-up measures six months after the intervention.

The two metanalyses confirmed and identified some problems or gaps we have tried to overcome in the present study. For this reason, in our study, we propose to deepen the assessment of EI training for senior managers, aiming to overcome most of the limitations mentioned in the studies of Kotsou et al. [55] and Hodzic et al. [8] by implementing the following: 1) Include a control group (waiting list group); 2) Conduct follow-up measurements (12 months later); 3) Employ an experimental design; 3) Include a workshop approach with group discussions and interactive participation; 4) Identify specific individual differences (i.e., age, gender) that might determine the effects of interventions; and 5) Use self-report and ability measures. For these reasons, two different ways of evaluating EI have been selected in this study to assess the emotional competencies applied within the labor and business world to solve practical problems: the EQ-i questionnaire [2], based on mixed models to provide a self-perceived index of EI, and the Situational Test of Emotional Management (STEM) and the Situational Test of Emotional Understanding (STEU) [56] based on the ability model. Thus, including two different EI measure we aim at obtaining a more reliable validation of the intervention used.

Therefore, the objective of our study was to investigate whether EI can be improved among employees who occupy senior management positions in a private company. Thus, the research hypothesis was that participation in the designed program would improve EI among senior managers.

### EI training development

The Course on Emotional Intelligence (TCEI) was created to provide senior managers with emotional knowledge and practical emotional skills so that they can apply and transfer their new understanding to teamwork and find solutions to real company problems and challenges. In this way, TCEI prepares workers to use the emotional learning resources appropriate to each work situation. In addition, TCEI combines face-to-face work sessions with a cross-sectional training through an e-learning platform. For more details, see S1 Appendix 1.

According to Mikolajczak [57], three interrelated levels of emotional intelligence can be differentiated: a) conceptual-declarative emotion knowledge, b) emotion-related abilities, and c) emotion-related dispositions. The TCEI aims at developing emotional skills, which are included on the second level of Mikolajczak’s model. Moreover, the present study uses the mixed model and the ability model measures to assess the level of EI. In using these measures, it is possible to assess the second level of Mikolajczak’s model. Pérez-González and Qualter [13] also suggest that activities related to ability EI should be included in emotional education programs.

Thus, this EI program was designed to allow senior managers to make use of their understanding and management of emotions as a strategy to assist them in facing the challenges within their work environment and managing their workgroups. Following the recommendation of Pérez-Gonzáles and Qualter, the training intervention methodology is founded in DAPHnE key practices [13]. It is important to emphasize that this training is grounded in practicality since it works based on the resolution of real cases, utilizing participative teaching-learning techniques and cooperative learning, while promoting the transfer of all aspects of EI and applied to various situations that can occur in the workplace. The e-learning system in the Moodle platform also provides an added value since it allows the creation of an environment providing exposure to professional experiences and continuous training. This type of pedagogical approach based on skills training and mediated through e-learning is a methodology that emerged in the 1990s when business organizations sought to create environments better suited to improving the management of large groups of employees. After its success, it began to be used in other contexts, including higher education and organizational development [58–60].

Finally, in order to justify the chosen training, it is important to note that the following official competencies for senior managers have been designated by the company:

1. Supervise the staff and guarantee optimum employee performance by fostering a motivational working environment where employees receive the appropriate support and respect and their initiatives are given the consideration they deserve.
2. Make decisions and promote clear goals, efficient leadership, competitive compensation, and acknowledgment of the employees’ achievements.
3. Justify their decisions to executives and directors, explaining how they have ensured training by creating opportunities for appropriate professional development for all employees and how they have facilitated conditions for a better balance in achieving the company’s objectives.

In conclusion, considering the above-mentioned professional competencies required, senior managers were selected as participants in this study since they need to possess and apply aspects related to EI in order to accomplish their leadership and staff management responsibilities.

## Method

### Participants

The company participating in this study was an international company with almost 175 years of history that occupies a select position in a branch of industry in the natural gas value chain, from the source of supply to market, including supply, liquefaction, shipping, regasification, and distribution. The company is present in over 30 countries around the world.

This study was conducted involving a sample of 54 senior managers selected from a company in a European country. The sample was extracted from the entire population of senior managers within this company following a stratified random sampling procedure, taking into account the gender of the population in order to select 50% of each gender.

The mean age of participants was 37.61 years (standard deviation = 8.55) and the percentage of female senior managers was 50%. For evaluation purposes, these employees were randomly divided into two groups: the experimental group (*n* = 26; mean age = 35.57 (7.54); 50% women) and the control group (*n* = 28; mean age = 39.50 (9.11); 50% women). The control group received EI training after the last data collection.

### Procedure

Initially, a group of senior managers from the company was selected to participate in the study, as they are employees who need a special domain of EI due to the competencies assigned to their professional category. In all cases, informed consent was requested for their participation in the study.

Assignment of participants to each condition, experimental or control, was performed using a random-number program. In addition, to avoid the Hawthorne effect, participants were not told if they were assigned to the experimental or control group; only their consent to participate in research on the development of EI was asked. Participants from the control group completed the same evaluations as the training group but were not exposed to the training.

The scales were administered during the pretest phase (Time 1) on an online platform for the experimental and control groups. On average, approximately 90 minutes were needed to complete the tests.

After the data were collected in the pre-test phase, only the experimental group participated in the TCEI over seven weeks, and they received a diploma.

Later, the scales were administered during the posttest phase (Time 2). Similarly, we collected the same data one year later (Time 3). A lapse of one year was allowed to pass because all training programs carried out in this company are re-evaluated one year later to determine whether improvements in employees’ skills were maintained over time. In fact, this demonstrates a clear commitment to monitoring the results achieved. Other studies have also used reevaluations of their results. For example, according to Nelis et al. [22] and Nelis et al. [39], the purpose of their studies was to evaluate whether trait EI could be improved and if these changes remained. To accomplish this, the authors performed three assessments: prior to the intervention, at the end of the intervention, and six months later. Therefore, as recommended by Kirkpatrick [61], research on the effectiveness of training should also include a long-term assessment of skills transfer.

Finally, is important to remark that all participants were properly informed of the investigation, and their written consent was obtained. All methods were performed in accordance with the relevant guidelines and regulations and the study was approved by University of Alicante Ethics Committee (UA-2015-07-06) and carried out in accordance with the relevant guidelines and regulations.

### Measures

As mentioned before, two different ways of defining and evaluating EI were selected for this study: (1) EQ-i, based on mixed models, and (2) the STEM/STEU questionnaires, based on the ability model of EI.

1 The Emotional Quotient Inventory [2]

To measure EI based on the mixed models, the short version of the EQ-i was used, which comprises 51 self-referencing statements and requires subjects to rate the extent to which they agree or disagree with each statement on a five-point Likert scale (1 = strongly disagree; 5 = strongly agree). An example item is the following; “In handling situations that arise, I try to think of as many approaches as I can.” The EQ-i comprises five factors: Intrapersonal EI and Self-Perception, Interpersonal EI, Adaptability and Decision Making, General Mood and Self-Expression, Stress Management, and a Total EQ-i score, which serves as a global EI measure. The author of this instrument reports a Cronbach’s alpha ranging from .69 to .86 for the 5 subscales [2, 62] and the Cronbach’s alpha of the Emotional Quotient Inventory was .80 for the present sample of senior manager.

2 Situational Test of Emotional Understanding (STEU) and Situational Test of Emotion Management (STEM) [63]

Two tests were used to measure EI based on the ability model. Emotion understanding was evaluated by the short version of the Situational Test of Emotional Understanding (STEU) [63]. This test is composed of 25 items that present an emotional situation (decontextualized, workplace-related, or private-life-related). For each item, participants have to choose which emotion will most likely elicit the described situation. Cronbach’s alpha of STEU is .83 [63] and the Cronbach’s alpha of the Situational Test of Emotional Understanding was .86 for the present sample of senior manager. An example item is the following: “An unwanted situation becomes less likely or stops altogether. The person involved is most likely to feel: (a) regret, (b) hope, (c) joy, (d) sadness, (e) relief” (in this case, the correct answer is “relief”).

On the other hand, emotion management was evaluated by the short version of the Situational Test of Emotion Management (STEM) [63]. This test is composed of a 20-item situational judgment test (SJT) that uses hypothetical behavioral scenarios followed by a set of possible responses to the situation. Respondents must choose which option they would most likely select in a “real” situation. Cronbach’s alpha of STEM is .68 [63] and the Cronbach’s alpha of the Situational Test of Emotion Management was .84 for the present sample of senior manager. An example item is the following: “Pete has specific skills that his workmates do not, and he feels that his workload is higher because of it. What action would be the most effective for Pete? (a) Speak to his boss about this; (b) Start looking for a new job; (c) Be very proud of his unique skills; (d) Speak to his workmates about this.”

### TCEI content and organization

The program schedule spanned seven weeks with a face-to-face session of 95 minutes each week, which was delivered by one of the researchers specifically trained for this purpose. All the experimental group participants were taught together in these sessions. The content of each session was the following:

*1st Session*: Introduction. The objectives and methodology of the training were explained to participants.

*2nd Session*: Intrapersonal EI and self-perception. Trainees learned to identify their own emotions.

*3rd Session*: Interpersonal EI. Participants learned to identify others’ emotions.

*4th Session*: Adaptability and decision making. The objective was to improve trainees’ ability to identify and understand the impact that their own feelings can have on thoughts, decisions, behavior, and work performance resulting in better decisions and workplace adaptability.

*5th Session*: General mood and self-expression. Trainees worked on expressing their emotions and improving their skills to effectively control their mood.

*6th Session*: Stress management. Participants learned EI skills to manage stress effectively.

*7th Session*: Emotional understanding and emotion management. Trainees learned skills to effectively manage their emotions as well as skills that influence the moods and emotions of others.

In addition, access to the virtual environment (Moodle platform) was required after each face-to-face session. The time spent in the platform was registered, with a minimum of five hours required per week.

The virtual environment allowed the researcher to review all the content completed in each face-to-face session.

All of the EI abilities included in the virtual part of the training have been previously used in the face-to-face part; thus, virtual training is simply a method used to consolidate EI knowledge. In fact, the virtual environment has the same function as completing a workbook about the information presented during the face-to-face session. However, the added advantage of working in an e-learning environment is that all of the trainers are connected and can share their tasks and progress with others. At times, in addition to reviewing the contents of the previous session, the e-learning environment also introduces some important terms for the next session utilizing the principles of the well-known flipped classroom methodology. In short, the following activities were carried out through the Moodle platform to consolidate the participants’ knowledge:

1st Session: Participants were informed that e-learning would be part of the training in order to consolidate EI knowledge.

2nd Session: Participants explored the skills of Intrapersonal EI and self-perception in the virtual environment through discussion forums.

3rd Session: Participants learned the skills of identifying others’ emotions and utilizing this emotional information for decision-making. This information was summarized in the virtual environment through discussion forums.

4th Session: Participants sharpened their skills of adaptability and decision-making through the production of innovative ideas and the utilization of critical thinking skills in assessing the impact that their own feelings can have on others’ work performance. Trainees learned how to express their own emotions, as well as the skill of effectively controlling their mood, through the resolution of practical cases in the virtual environment; in these cases, innovative ideas and critical thinking skills were required in order to make better decisions during emotionally impactful; situations. In addition, trainees utilized the forum to reflect on why their own emotional regulation is important for ensuring long-term workplace adaptability.

5th Session: Verbal quiz, discussion, and forum contribution. Trainees participated in an online debate about key emotional skills in order to understand how to apply them in a real work environment. In particular, the debate focused on regulating the self-expression skill and equilibrating the general mood when there are difficult situations within the company. In this way, the participants identified the skills required to effectively manage the stress experienced in order to maintain a positive mood A discussion about common stressful situations at work was carried out in the virtual environment, and strategies for regulating the mood during critical work situations were shared.

6th Session: Discussion of ideas related to EI. Trainees participated in an online debate about key emotional skills in order to understand how to apply stress management skills to the real work environment. It was necessary to share previous work experiences where stress was a significant challenge and reevaluate the emotionally intelligent way to deter stress and maintain a balanced senior manager life.

7th Session: Participants concluded the training on target strategies to effectively manage their emotions as well as skills that influence the moods and emotions of others. This session, therefore, was a period for feedback where brief answers to specific doubts were provided. In addition, the outcomes of the training were established by the participants. Finally, senior managers were encouraged to stay connected through the Moodle platform in order to resolve future challenges together using the EI skills learned and internalized during the training period.

### Data analysis

An experimental pretest-posttest with a control group design was adopted. Under this design, multivariate variance analysis (MANOVA) and univariate variance analysis (ANOVA) of repeated measures were performed, in which the measures of dependent variables were treated as variables evaluated within the same subjects, and groups operated as variables between subjects. Finally, all statistical analyses were conducted using SPSS statistical software, version 21.0 (IBM, Armonk, USA).

## Results

First, sample normality analysis indicated that the population followed a normal distribution. The results of Box’s M test did not show homogeneity in the variance-covariance matrix on the EQ-i Total Scale (M = 59.29; *F* = 9.26, *p* ≥ 0.00) or on the STEM/STEU (M = 231.01; *F* = 36.07, *p* ≥0.00). However, Hair et al. [64] have stated that if the control and the experimental groups are of equal size, which was the case in this study, then that factor tends to mitigate the effects of violations of the normality assumption.

Second, to test whether there was any significant difference between the experimental group and control group at the time of pretest, Student’s *t*-test was performed to determine the differences in means of all the variables measured (Table 1). Table 1 shows that there were no significant differences at the time of pretest. This finding suggests that both groups began in analogous situations.

**Table 1:** Student’s t-test of differences in means (t1, t2, t3).

	*X(SD)*	*X(SD)*	*t*	*gl*	Sig.	Difference	*SD*	95% Confidence	
	*Control Group*	*Experimental Group*						Lower	Upper
1. Age	.22 (1.07)	-.24 (.88)	1.71	52	.09	.45	.26	-.07	.99
	26.67 (6.18)^1^	25.00 (4.77)^1^	1.71^1^	52^1^	.09^1^	3.92^1^	2.28^1^	-.66^1^	8.51^1^
2. Intrapersonal (t1)	-.03 (1.12)	.03 (.87)	-.21	52	.83	-.05	.27	-.60	-.49
	24.67 (6.18) ^1^	25.00 (4.77) ^1^	-.21^1^	52^1^	.83^1^	-.32^1^	1.51^1^	-3.35^1^	2.71^1^
3. Interpersonal (t1)	.19 (1.07)	-.21 (.89)	1.49	52	.14	.40	.26	-.13	.94
	39.50 (3.28) ^1^	38.26 (2.70) ^1^	1.49^1^	52^1^	.14^1^	1.23^1^	.82^1^	-.41^1^	2.88^1^
4. Stress Man. (t1)	-.03 (1.07)	.03 (.94)	-.24	52	.80	-.06	.27	-.61	.48
	20.35 (5.97) ^1^	20.73 (5.29) ^1^	-.24^1^	52^1^	.80^1^	-.37^1^	1.53^1^	-3.45^1^	2.70^1^
5. Adaptability (t1)	-.04 (1.04)	.05 (.98)	-.33	52	.73	-.09	.27	-.64	.45
	28.46 (3.85) ^1^	28.80 (3.64) ^1^	-.33^1^	52^1^	.73^1^	-.34^1^	1.02^1^	-2.39^1^	1.70^1^
6. General Mood (t1)	.12 (.90)	-.13 (1.10)	.90	52	.36	.24	.27	-.29	.79
	35.25 (2.02) ^1^	34.69 (2.47) ^1^	.90^1^	52^1^	.36^1^	.55^1^	.61^1^	-.67^1^	1.79^1^
7. Total EQi (t1)	.05 (.99)	-.05 (1.03)	.34	52	.73	.09	.27	-.45	.64
	29.65 (1.57) ^1^	29.50 (1.64) ^1^	.34^1^	52^1^	.73^1^	.15^1^	.43^1^	-.72^1^	1.02^1^
8. STEU (t1)	.06 (1.03)	-.06 (.98)	.41	52	.67	.11	.27	-.43	.66
	13.28 (2.91) ^1^	12.96 (2.79) ^1^	.41^1^	52^1^	.67^1^	.32^1^	.77^1^	-1.23^1^	1.88^1^
9. STEM (t1)	.06 (1.00)	-.07 (1.01)	.48	52	.62	.13	.27	-.41	.68
	11.93 (.97) ^1^	11.80 (.98) ^1^	.48^1^	52^1^	.62^1^	.13^1^	.26^1^	-.40^1^	.66^1^
10. Intrapersonal (t2)	-.79 (.71)	.85 (.36)	-10.78	52	.00	-1.63	.15	-1.94	-1.32
	23.53 (5.98) ^1^	37.34 (3.05) ^1^	-10.78^1^	52^1^	.00^1^	-13.81^1^	1.28^1^	-16.39^1^	-11.22^1^
11. Interpersonal (t2)	-.21 (1.01)	.22 (.96)	-1.59	52	.11	-.42	.26	-.96	.11
	39.82 (2.88) ^1^	41.03 (2.72) ^1^	-1.59^1^	52^1^	.11^1^	-1.21^1^	.76^1^	-2.74^1^	.31^1^
12. Stress Man. (t2)	-.68 (.79)	.73 (.61)	-7.25	52	.00	-1.40	.19	-1.79	-1.01
	18.10 (5.64) ^1^	28.11 (4.35) ^1^	7.25^1^	52^1^	.00^1^	-10.00^1^	1.38^1^	-12.77^1^	-7.23^1^
13. Adaptability (t2)	.29 (1.01)	-.31 (.91)	2.30	52	.02	.60	.26	.07	1.12
	29.78 (3.68) ^1^	27.57 (3.32) ^1^	2.30^1^	52^1^	.02^1^	2.20^1^	.95^1^	.28^1^	4.13^1^
14. General Mood (t2)	-.65 (.74)	.70 (.75)	-6.64	52	.00	-1.34	.20	-1.74	-.93
	35.50 (2.44) ^1^	39.96 (2.48) ^1^	-6.64^1^	52^1^	.00^1^	-4.46^1^	.67^1^	-5.80^1^	-3.11^1^
15. Total EQi (t2)	-.85 (.60)	.92 (.20)	-14.71	52	.00	-1.76	.12	-2.01	-1.52
	29.35 (1.85) ^1^	34.80 (.61) ^1^	-14.71^1^	52^1^	.00^1^	-5.45^1^	.37^1^	-6.21^1^	-4.70^1^
16. STEU (t2)	-.67 (.98)	.73 (.17)	-7.45	52	.00	-1.40	.18	-1.78	-1.01
	13.28 (2.91) ^1^	14.46 (.50) ^1^	-7.45^1^	52^1^	.00^1^	-4.17^1^	.56^1^	-5.32^1^	-3.02^1^
17. STEM (t2)	-.68 (.98)	.73 (.15)	-7.50	52	.00	-1.40	.18	-1.78	-1.02
	11.96 (.96) ^1^	13.34 (.14) ^1^	-7.50^1^	52^1^	.00^1^	-1.38^1^	.18^1^	-1.75^1^	-1.00^1^
18. Intrapersonal (t3)	-.77 (.75)	.83 (.34)	-10.26	52	.00	-1.60	.15	-1.92	-1.29
	25.57 (5.73) ^1^	37.84 (2.57) ^1^	-10.26^1^	52^1^	.00^1^	-12.27^1^	1.19^1^	-14.69^1^	-9.85^1^
19. Interpersonal (t3)	-.24 (.96)	.26 (.99)	-1.88	52	.06	-.50	.26	-1.03	.03
	39.57 (3.47) ^1^	41.38 (3.57) ^1^	-1.88^1^	52^1^	.06^1^	-1.81^1^	.96^1^	-3.74^1^	.11^1^
20. Stress Man. (t3)	-.71 (.75)	.76 (.59)	-7.90	52	.00	-1.46	.18	-1.83	-1.09
	19.14 (5.01) ^1^	28.88 (3.93) ^1^	-7.90^1^	52^1^	.00^1^	-9.74^1^	1.23^1^	-12.21^1^	-7.26^1^
21. Adaptability (t3)	-.05 (1.18)	.06 (.78)	-.38	52	.69	-1.06	.27	-.65	.44
	27.96 (4.22) ^1^	28.34 (2.78) ^1^	-.38^1^	52^1^	.69^1^	-.38^1^	.98^1^	-2.35^1^	1.58^1^
22. General Mood (t3)	-.63 (.80)	.68 (.72)	-6.34	52	.00	-1.31	.20	-1.72	-.89
	35.53 (2.54) ^1^	39.73 (2.29) ^1^	-6.34^1^	52^1^	.00^1^	-4.19^1^	.66^1^	-5.52^1^	-2.86^1^
23. Total EQi (t3)	-.87 (.55)	.93 (.21)	-16.16	52	.00	-1.80	.11	-2.02	-1.57
	29.55 (1.73) ^1^	35.23 (.74) ^1^	-16.16^1^	52^1^	.00^1^	-5.68^1^	.35^1^	-6.39^1^	-4.26^1^
24. STEU (t3)	-.75 (.86)	.81 (.15)	-9.46	52	.00	-1.55	.16	-1.89	-1.21
	13.35 (2.89) ^1^	18.61 (.49) ^1^	-9.46^1^	52^1^	.00^1^	-5.25^1^	.55^1^	-6.39^1^	-4.12^1^
25. STEM (t3)	-.79 (.74)	.85 (.27)	-11.01	52	.00	-1.64	.14	-1.94	-1.34
	11.99 (.94) ^1^	14.09 (.34) ^1^	-11.01^1^	52^1^	.00^1^	-2.09^1^	.19^1^	-2.48^1^	-1.71^1^

Note. t1 = pretest; t2 = posttest; t3 = follow-up.

^1^ = direct score

Therefore, we came to the conclusion that the two groups of workers could not be distinguished by EI level before the TCEI program. In addition, the mean age of each group was analyzed and no baseline differences were found between the two groups.

To assess the impact of the program on EI, the scores obtained by both groups were compared before its implementation (pretest–Time 1) and shortly after the program was delivered (posttest–Time 2), as well as one year later (follow-up–Time 3). Group membership was the independent factor or variable, and the scores obtained by the subjects regarding EI were the criteria or dependent variables.

Two control variables, gender and age, were included in the analysis because they could affect the results. However, none of these variables showed a statistically significant effect in any of the variables assessed (p≥ .50 in all cases).

Regarding the implementation of the program, Table 2 presents the test results for intra-subject effects, which showed significant Group x Time interaction for all variables except for Adaptability.

**Table 2:** Summary of intra- and inter-subject univariate ANOVA.

	Source	TypeIII	*df*	*F*	*p*	η^2^partial	Ob.Power
Intrapersonal	Intra	1441.19	2	4.27	< .01	.44	1.00
	Intra*Inter	147.35	2	41.09	< .01	.44	1.00
	Error intra	186.95	104				
	Inter	3133.58	1	81.63	< .01	.61	1.00
	Error inter	1996.25	52				
Interpersonal	Intra	88.61	2	4.59	.01	.08	.77
	Intra*Inter	7.16	2	3.64	.03	.07	.66
	Error Intra	1003.46	104				
	Inter	14.55	1	1.44	.24	.03	.22
	Error inter	526.74	52				
Stress Management	Intra	349.53	2	1.33	< .01	.17	.99
	Intra*Inter	811.85	2	23.99	< .01	.32	1.00
	Error Intra	1759.69	104				
	Inter	1819.82	1.00	41.23	< .01	.44	1.00
	Error inter	2295.26	52				
Adaptability	Intra	9.17	2	.44	.65	.01	.12
	Intra*Inter	59.44	2	2.85	.06	.05	.55
	Error Intra	1.084.30	104				
	Inter	9.89	1	.53	.47	.01	.11
	Error inter	968.61	52				
General Mood Scale	Intra	264.45	2	22.41	< .01	.30	1.00
	Intra*Inter	215.04	2	18.23	< .01	.26	1.00
	Error Intra	613.54	104				
	Inter	294.76	1	56.09	< .01	.52	1.00
	Error inter	273.29	52				
Total EQi	Intra	257.75	2	206.82	< .01	.80	1.00
	Intra*Inter	294.35	2	236.19	< .01	.82	1.00
	Error Intra	64.80	104				
	Inter	542.67	1.00	107.11	< .01	.67	1.00
	Error inter	263.46	52				
STEU	Intra	245.05	2	131.15	< .01	.72	1.00
	Intra*Inter	236.31	2	126.47	< .01	.71	1.00
	Error Intra	97.16	104				
	Inter	372.94	1	24.35	< .01	.32	1.00
	Error inter	796.27	52				
STEM	Intra	38.75	2	188.88	< .01	.78	1.00
	Intra*Inter	34.91	2	17.18	< .01	.77	1.00
	Error Intra	1.67	104				
	Inter	5.46	1	28.59	< .01	.35	1.00
	Error inter	91.76	52				

The observed power was highest in the key scales: 1.00 for the STEU/STEM and Total EQ-i. Regarding the subscales, the observed power was also 1.00 for the Intrapersonal, Stress Management, and General Mood subscales; on the other hand, the observed power for the Interpersonal and the Adaptability subscales was .66 and .55, respectively.

Similarly, the effect size (η^2^), the proportion of total variability attributable to a factor, and the magnitude of the difference between one time and another resulting from the interaction between the time of assessment and implementation of the program, was high for the key scales: ≥.71 for the STEU/STEM, and .82 for the Total EQ-i. With regards to the subscales, the effect size (η^2^) was the following: .44 for Intrapersonal, .07 for Interpersonal, .32 for Stress Management, .05 for Adaptability, and .26 for General Mood.

To further explain these results, complementary analyses were performed. On the one hand, as shown in Table 1, we carried out an average comparison between the experimental and control groups at the measurement moments T2 and T3. Results revealed significant differences between the experimental group and the control group regarding all variables and in both moments (T2 and T3), except for the Interpersonal variable, in which the experimental group obtained higher scores in these two moments but without being statistically significant these differences. This could explain the small effect size obtained for this variable.

In addition, the Adaptability variable showed statistically significant differences between the experimental group and the control group at time T2, with the control group scoring higher, while at time T3, the experimental group also obtains higher scores regarding Adaptability; however, this score difference with regards to the control group was not statistically significant. This could explain why the interaction was not significant and the small effect size obtained for this variable.

In order to compare differences between moments T1, T2, and T3, the marginal means were analyzed for both groups (experimental and control) per moment and variable (Table 3).

**Table 3:** Marginal means comparing t1-t2, t1-t3, and t2-t3.

variable	t1-t2			t1-t3			t2-t3			
	Mean dif	*p*	CI 95%	Mean dif	*p*	CI 95%	Mean dif	*p*	CI 95%	Summary differences
	(sd)			(sd)			(sd)			t1, t2, t3
Intrapersonal (EG)	-0.82 (.19)	< .00	-1.31/-.33	.80 (.15)	< .00	-1.20/-.41	-.02 (.10)	>1	.28/.25	t3>t1<t2 = t3
Interpersonal (EG)	-.43 (.27)	>.36	-1.12/.26	-.47 (.29)	>.35	-1.21/.27	.04 (.27)	>1	.65/.73	t3 = t1 = t2 = t3
Stress Management (EG)	-.69 (21)	< .01	1.23/.16	-.73 (.23)	< .01	-1.31/-.14	.03 (.16)	>1	-.38/.44	t3>t1<t2 = t3
Adaptability (EG)	.36 (.22)	>.34	-.20/.93	-.01 (.23)	>1	-.60/.59	.37 (.23)	>.37	-.22/.96	t3 = t1 = t2 = t3
General Mood (EG)	-.83 (23)	< .03	-1.58/-.07	-.81 (.26)	< .01	-1.46/-.15	-.02 (.21)	>1	-.55/.52	t3>t1<t2 = t3
Total EQ-i (EG)	-.97 (.20)	< .00	-1.49/-.45	-.98 (.18)	< .00	-1.46/-.51	.02 (.06)	>1	-.13/.16	t3>t1<t2 = t3
STEU (EG)	-.79 (.17)	< .00	-1.21/-.36	-.87 (.17)	< .00	-1.30/-.43	.08 (.02)	< .01	.02/.14	t3>t1<t2<t3
STEM (EG)	-.80 (.17)	< .00	-1.25/-.35	-.92 (.15)	< .00	-1.31/-.53	.12 (.03)	< .00	.06/.19	t3>t1<t2<t3
Intrapersonal (CG)	.76 (.18)	< .00	.30/1.22	.75 (.23)	< .01	.17/1.32	.01 (.17)	>1	-.42/.45	t3<t1<t2 = t3
Interpersonal (CG)	.40 (.25)	>.36	-.24/1.04	.44 (.29)	>.42	-.30/1.17	-.04 (.22)	>1	-.61/.54	t3 = t1 = t2 = t3
Stress Management (CG)	.65 (.16)	< .00	.23/1.06	.67 (.17)	< .00	.24/1.11	-.03 (.15)	>1	-.41/.36	t3<t1>t2 = t3
Adaptability (CG)	-.34 (.20)	>.33	-.85/.18	.01 (.28)	>1	-.70/.71	-.34 (.27)	>.65	-1.03/.35	t3 = t1 = t2 = t3
General Mood (CG)	.77 (.21)	< .00	.24/1.29	.75 (.24)	< .01	.13/1.37	.02 (.19)	>1	-.48/.51	t3<t1>t2 = t3
Total EQ-i (CG)	.90 (.10)	< .00	.65/1.15	.91 (.10)	< .00	.65/1.18	-.02 (.05)	>1	-.15/.12	t3<t1>t2 = t3
STEU (CG)	.73 (.01)	< .00	.71/.75	.80 (.04)	< .00	.71/.90	-.08 (.03)	< .04	-0.15/.00	t3<t1>t2>t3
STEM (CG)	.74 (.01)	< .00	.71/.77	.86 (.05)	< .00	.73/.99	-.12 (.05)	< .05	-.23/.00	t3<t1>t2 = t3

Note. EG = experimental group; CG = control group; t1 = pretest; t2 = posttest; t3 = follow-up.

In general, in the experimental group, there was a significant improvement between moments T1 and T2 in all variables, except Interpersonal and Adaptability, which did not present changes at any of the three moments (T1, T2, T3). On the other hand, scores remained without significant changes regarding all variables between moments T2 and T3, except in the case of STEU and STEM, in which the scores continued to improve between moments T2 and T3.

In the control group, the results were the same as in the experimental group concerning the Interpersonal and Adaptability variables. However, with regards to other variables, the trend was inverse to the experimental group between moments T1 and T2; in this case, there was a significant decrease in the scores between these two moments in the rest of the variables. Between moments T2 and T3, the scores remained without significant changes in all the variables measured with the EQ-i. In the case of variables measured with the ability test, there was a significant decrease in the STEU scores between moments T2 and T3, whereas the STEM scores remained without significant changes.

**Fig 1:**
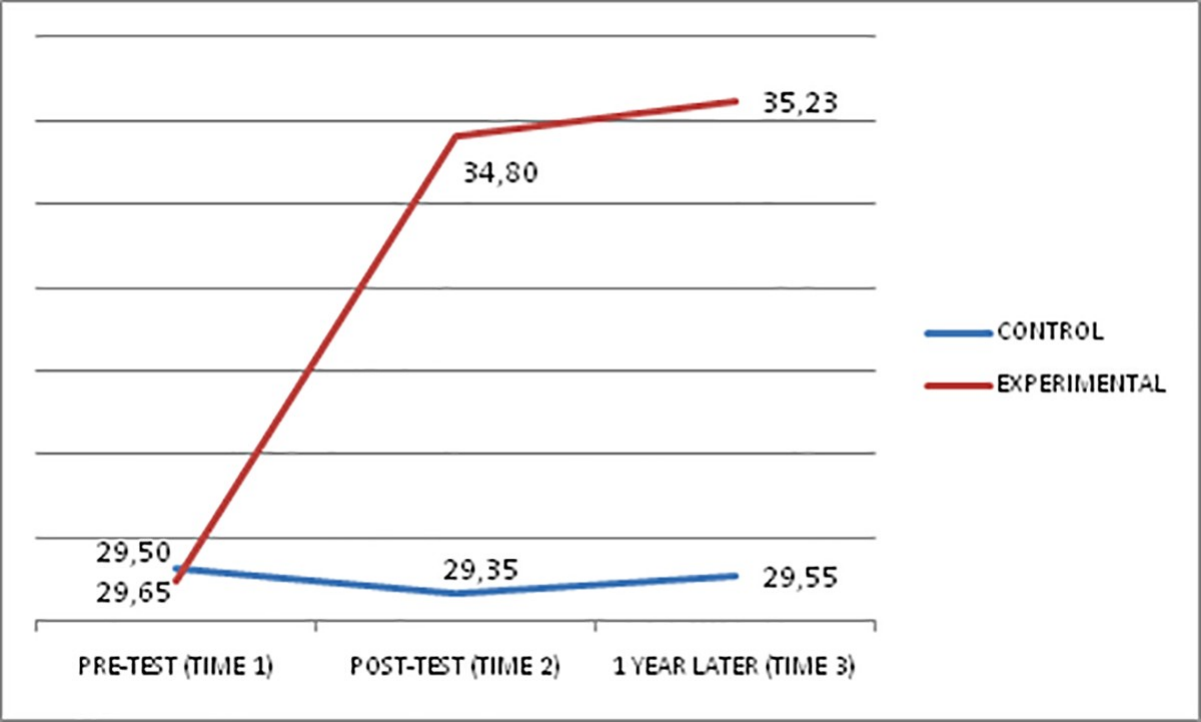
Total EQi performance of the groups at pre-test (Time 1), post-test (Time 2), and one year after (Time 3).

**Fig 2:**
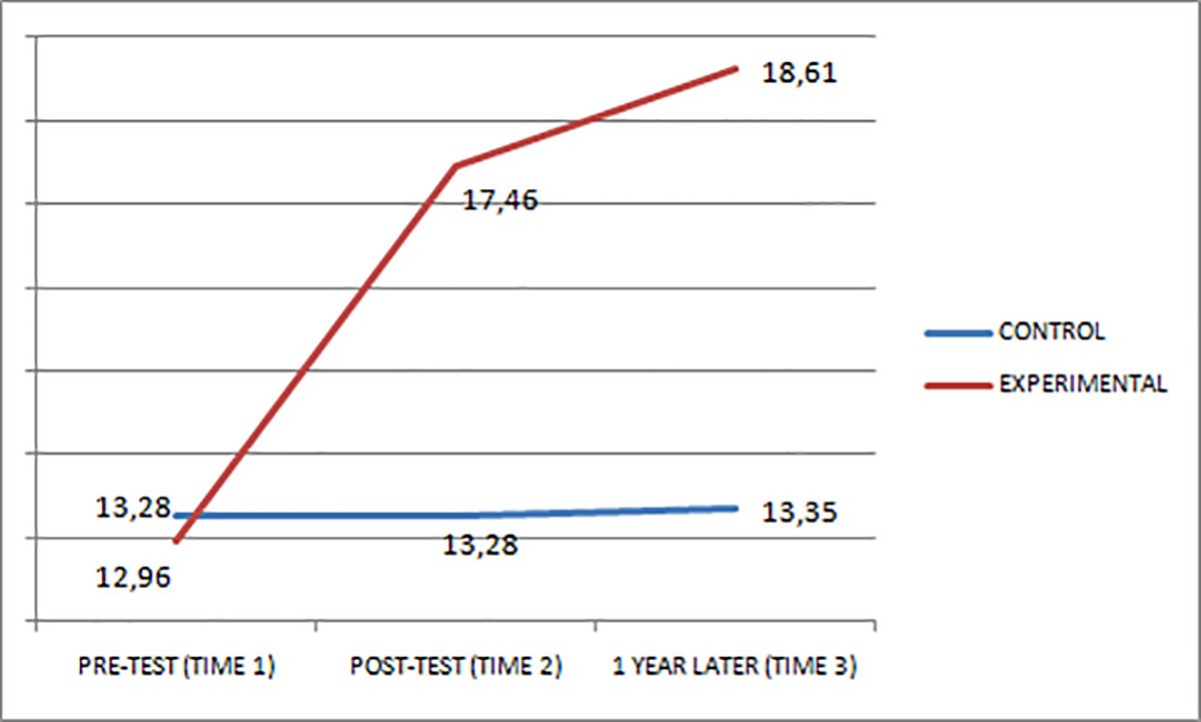
STEU performance of the groups at pre-test (Time 1), post-test (Time 2), and one year after (Time 3).

**Fig 3:**
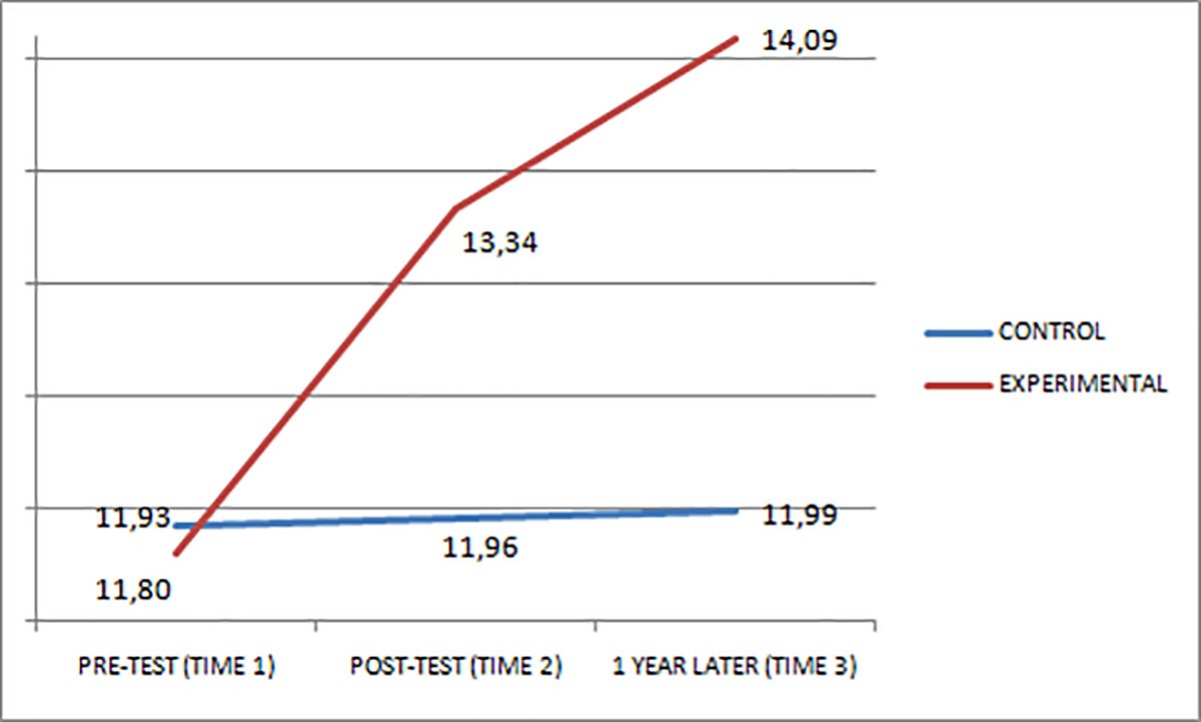
STEM performance of the groups at pre-test (Time 1), post-test (Time 2), and one year after (Time 3).

Figs 1–3 show the scores obtained in the EQ-i total scale and STEM/STEU total scales by both groups at Times 1, 2, and 3. At Times 2 and 3, the experimental group, which had received the EI training, had an increase in its scores, whereas the control group did not present any substantial change in scores.

## Discussion

The objective of this study was to examine the effectiveness of an EI training program among the senior managers (N = 54) of a private company. Consistent with Pérez-González and Qualter [13], Hodznik et al. [8], and Kotsou et al.’s [55] recommendations, we aimed to contribute new research findings and extend the existing literature on the effectiveness of EI training in the workplace. The main findings of this study revealed that intrapersonal EI, self-perception, general mood, self-expression, and stress management were maintained after the completion of the training. On the other hand, improvements in emotional understanding and emotion management had strengthened over time. However, the results also revealed that training did not result in similar improvements across all variables. Specifically, training had a nonsignificant impact on interpersonal and adaptability skills.

### Theoretical implications of the study

With regard to the theoretical implications of the present findings, the observed effectiveness of the TCEI, which was conducted using an innovative methodology that entailed face-to-face training and a virtual campus support system among senior managers, extends the existing literature on the development of EI training programs.

The training program that was conducted as a part of this study failed to improve two dimensions of EI: interpersonal and adaptability skills. There are two possible explanations for why these variables did not demonstrate improvement. First, high-quality training that addresses all the dimensions of EI is necessary to produce large effects. Therefore, the time and exercises that are devoted to these two dimensions of EI may need to be redefined. Accordingly, the second and fourth sessions of this training (i.e., interpersonal and adaptability skills, respectively) can be enriched by adding new activities and including long-term evaluation of the transfer of skills to real workplace situations in which these abilities are required to resolve challenges. Indeed, allocating more time and exercises to these topics may have offered participants greater experience in practicing these interpersonal and adaptability skills in regular and virtual classroom settings before applying them in the workplace.

On the other hand, changes in these two dimensions of EI may not be detectable immediately after the completion of the training or soon after a year has elapsed. Similarly, the studies that Kotsou et al. reviewed [55] also indicated that improvements in EI may not be detectable immediately or shortly after the completion of an intervention. Further, the conclusions of this review appear to suggest that shorter training programs do not improve some dimensions of EI. Therefore, a more intensive training and longer time gap between completion of training and assessment (i.e., after more than a year has elapsed) may yield significant results for these two dimensions of EI. Indeed, other studies have used longer time gaps such as more than two years [40] and yearly evaluations across three years [47].

In any case, the present findings suggest that the proposed training intervention is effective in improving some dimensions of EI. In particular, senior managers who received EI training demonstrated significant improvements in their ability to perceive, understand, and accept their own and others’ emotions in an effective way, be self-reliant, achieve personal goals, manage stress, have a positive attitude, and control and manage emotions; these findings are consistent with those of past studies that have aimed to improve EI by providing training in workplaces [45–52].

The largest effects emerged for the total scores for EI (as per mixed models; total EQ-i), followed by emotion management (STEM) and understanding (STEU), intrapersonal aspects, stress management, and finally, general mood. Moreover, improvements in emotional understanding and emotion management that had resulted from the training intervention had strengthened over time.

Similarly, several researchers have indicated that EI plays a key role in leadership development and success in the workplace [65, 66]. The behaviors of managers shape critical stages of their subordinates’ careers as well as the provision of optimal training and promotion [67, 68]. Given the unique significance that EI and optimal leadership bears to this group of professionals, the present study aimed to improve the EI of senior managers.

In sum, the proposed program is a training intervention that can be used to enhance the EI of senior managers because, as the previously articulated extensive literature review has demonstrated, EI plays a key role within work environments. Therefore, the present findings suggest that the TCEI is an effective training program that can improve the ability to identify one’s own and others’ emotions as well as identify and understand the impact of one’s feelings on thoughts, decisions, behaviors, and performance at work.

### Practical implications

The present findings serve as empirical evidence of the effectiveness of the training program that was conducted in the present study in improving key dimensions of EI that foster the emotional skills that are both necessary and desirable in the workplace. Accordingly, the present findings have practical implications because they support the future use of the EI training program that was used in the present study. In this regard, the present findings revealed that EI training can promote the emotional development of senior managers.

In addition, the methodology of the training program is noteworthy because it required participants to use communication and work as a group to solve real practical problems that necessitate the application of EI skills in the workplace. Similarly, the use of face-to-face training alongside an e-learning platform helped participants acquire the ability to learn independently as well as synergically (i.e., with other senior managers). This encouraged the group to reflect on their knowledge about EI and apply their EI skills to handle workplace challenges.

It is important to emphasize that there were significant temporal changes in the scores of measures of emotional understanding and emotion management; in other words, the scores continued to improve a year after the completion of training. It is interesting to note that the methodology of the last training session was unique because it involved the creation of a “life and career roadmap” and “commitment to growth and development. We believe that these exercises were responsible for the continued improvement in important EI skills over time that was observed in the present study.

This finding has important practical implications because it underscores the importance of requiring senior managers to indicate their commitment to the transfer of knowledge. Indeed, the roadmap defines the results that are expected to follow the implementation of the learned emotional strategies and verifies the achievement of these results. In addition, all managers signed an online contract to indicate their commitment to remain connected through the virtual campus support system to resolve any conflicts that may arise within the company in an emotionally intelligent manner.

We believe that the method of learning that our intervention entailed is more effective than conventionally used methods. Further, the uniqueness of this method may have contributed to the observed change in scores because it allowed frustrated senior managers to share their unresolved issues. Finally, by practicing emotional understanding and emotional management during the training, the created a plan of action and implemented their solutions using EI strategies.

In addition, we believe that signing the online contract helped them understand their responsibilities and the impact that their emotional understanding and emotion management can have on the organization. The fact that their scores on measures of emotional understanding and emotion management continued to increase over time indicates that the subjects had acquired these skills and that, once they had acquired them, they continued to develop them. Similarly, Kotsou et al. [55] also found that training resulted in stable improvements in EI. In addition to providing their participants with EI tools and skills as a part of their training, they also motivated them to apply these skills and use these tools in the future.

Taken together, the present findings have promising practical implications. Specifically, the findings suggest that a training methodology that facilitates knowledge transfer (i.e., application of knowledge about EI in the management of workplace challenges) can enhance the following dimensions of EI: emotional understanding, emotion management, self-perception (through training activities that pertain to self-regard, self-actualization, and emotional self-awareness), decision making (through training activities that pertain to problem solving, reality testing, and impulse control), self-expression (through training activities that pertain to emotional expression, assertiveness, and independence), and stress management (through training activities that pertain to flexibility, stress tolerance, and optimism).

### Limitations and future studies

The present study has several limitations that require explication. First, we included only age and gender as control variables and omitted other individual differences that could have influenced the results. However, it is important for future researchers to define and examine the role of individual differences in the effects of EI training in greater detail. In addition, in accordance with Kotsou et al. [55] and Hodzic et al.’s [8] suggestions, detailed behavioral indicators must be examined because they may play a crucial role in the effectiveness of EI training. Another limitation of the present study is that the intervention program was conducted in only one company. Therefore, future studies must implement this program in different companies and across varied business contexts. The present results make it apparent that further refinements are needed in order to address the aforementioned limitations of this intervention.

Another limitation of the present study is that it did not assess the effect that improvements in EI can have on other variables. Accordingly, recommendations for further research include the determination of whether improvements in EI that result from training lead to improvements in other variables such as job satisfaction and performance and successful leadership, in accordance with the results of other research studies [69–72]. Thus, future research studies must consider these possibilities when they examine whether the TCEI has the potential to produce all the aforementioned outcomes at an organizational level. Furthermore, the intervention can be redesigned in such a manner that it yields specific performance outcomes. Further, longitudinal studies on the effectiveness of EI training must be conducted across several sectors and countries.

Finally, senior managers define and direct the careers of the rest of a company’s personnel; Therefore, future research studies must examine how EI training can be used to promote its previously observed desirable effects such as the demonstration of good leadership behaviors, effective cooperation, and teamwork [29, 31, 34–38, 69]. In fact, this is an interesting line of inquiry for future researchers.

## Conclusions

In conclusion, the present findings contribute to the existing knowledge on the development of EI because they indicate that the training program resulted in improvements in many dimensions of the EI of senior managers. More specifically, the longitudinal effects of EI training on senior managers’ emotional skills had maintained over time, whereas the corresponding effects on emotional understanding and emotion management had strengthened at one-year follow up. Finally, the implementation of this intervention in organizational settings can nurture and promote a sense of fulfillment among employees.

## Supporting information

S1 File DATOS.sav. Data underlying the findings described.
(DOCX)

S2 File Appendix 1. TCEI planning schedule.
(SAV)
