# Supplementary material for: Can emotional intelligence be improved? A randomized experimental study of a business-oriented EI training program for senior managers
Source: PLoS One. 2019 Oct 23;14(10):e0224254. doi: 10.1371/journal.pone.0224254 (PMC6808549; doi:10.1371/journal.pone.0224254)
Supplement: S1 File — Data underlying the findings described. (DOCX) [file pone.0224254.s001.docx]

**Appendix** 1. TCEI planning schedule

| WEEK | TRADITIONAL TEACHING METHOD | TARGET | E-LEARNING TEACHING ON VIRTUAL PLATFORM |
| --- | --- | --- | --- |
| 1 | 1st Session:  The objectives and methodology of the training were explained  . | Introduction | 1st Session: E-learning method was explained. |
| 2 | 2nd Session:  Intrapersonal EI and  Self-Perception | Self-Regard  Self-Actualization  Emotional Self-Awareness | 2nd Session: Discussion about the skills of intrapersonal EI and self-perception through summarizing forums. |
| 3 | 3rd Session:  Interpersonal EI | Interpersonal Relationships  Empathy  Social Responsibility | 3rd Session: Discussion about the interpersonal EI skills through discussion forums. |
| 4 | 4th Session:  Adaptability and  Decision-Making | Problem Solving  Reality Testing  Impulse Control | 4th Session: Discussion about the skills of adaptability and decision-making through the production of innovative ideas and critical thinking skills in the resolution of real-life EI cases in the virtual environment. |
| 5 | 5th Session:  General Mood and  Self-Expression | Emotional Expression  Assertiveness  Independence | 5th Session: Verbal quiz, discussion, and forum contribution about General Mood and Self-Expression. |
| 6 | 6th Session:  Stress Management | Flexibility  Stress Tolerance  Optimism | 6th Session: Discussion of innovating ideas related to Stress Management through a brief pitch on the discussion forum. |
| 7 | 7th Session:  Emotional Understanding &  Emotion Management | Creating a Life and Career Roadmap & Developing a Commitment to Growth and Development | 7th Session: Feedback and conclusions of EI skills learned and internalized. Keep in touch via virtual campus. |
